# 1-(4a,8-Dimethyl-1,2,3,4,4a,5,6,8a-octa­hydro­naphthalen-2-yl)-3-(4-methyl­phen­yl)prop-2-en-1-one

**DOI:** 10.1107/S1600536811016941

**Published:** 2011-05-11

**Authors:** Mohamed Tebbaa, Ahmed Benharref, Moha Berraho, Daniel Avignant, Abdelghani Oudahmane, Mohamed Akssira

**Affiliations:** a Laboratoire de Chimie Bioorganique et Analytique, URAC 22, BP 146, FSTM, Université Hassan II, Mohammedia-Casablanca 20810 Mohammedia, Morocco; b Laboratoire de Chimie Biomoleculaire, Substances Naturelles et Réactivité, URAC16, Université Cadi Ayyad, Faculté des Sciences Semlalia, BP 2390, Boulevard My Abdellah, 40000 Marrakech, Morocco; c Université Blaise Pascal, Laboratoire des Matériaux Inorganiques, UMR CNRS 6002, 24 Avenue des Landais, 63177 Aubière, France

## Abstract

The title compound, C_22_H_28_O, was isolated from the aerial part of *Inula viscosa­* (L) Aiton [or *Dittrichia viscosa­* (L) Greuter]. The cyclo­hexene ring has a half-chair conformation, whereas the cyclo­hexane ring displays a chair conformation being substituted at position 2 by a 3-(4-methyl­phen­yl)prop-2-enoyl group. In the crystal, weak inter­molecular C—H⋯O hydrogen bonds link mol­ecules into chains in the [010] direction.

## Related literature

For background to the medicinal inter­est in *Inula viscosa­* (L) Aiton [or *Dittrichia viscosa­* (L) Greuter], see: Shtacher & Kasshman (1970 ▶); Bohlmann & Gupta (1982 ▶); Azoulay *et al.* (1986 ▶); Bohlmann *et al.* (1977 ▶); Ceccherelli *et al.* (1988 ▶). For details of the synthesis, see: Kutney & Singh (1984 ▶). For conformational analysis, see: Cremer & Pople (1975 ▶).
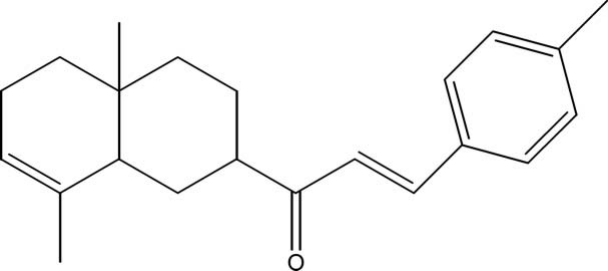


## Experimental

### 

#### Crystal data

C_22_H_28_O
*M* *_r_* = 308.44
Monoclinic, 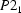

*a* = 7.1577 (2) Å
*b* = 10.3456 (2) Å
*c* = 12.3663 (3) Å
β = 95.557 (1)°
*V* = 911.43 (4) Å^3^
*Z* = 2
Mo *K*α radiation
μ = 0.07 mm^−1^
*T* = 298 K
0.37 × 0.16 × 0.16 mm

#### Data collection

Bruker X8 APEXII CCD area-detector diffractometer
8379 measured reflections
1957 independent reflections
1834 reflections with *I* > 2σ(*I*)
*R*_int_ = 0.020

#### Refinement

*R*[*F* ^2^ > 2σ(*F* ^2^)] = 0.039
*wR*(*F* ^2^) = 0.102
*S* = 1.11
1957 reflections
212 parameters
1 restraint
H-atom parameters constrained
Δρ_max_ = 0.16 e Å^−3^
Δρ_min_ = −0.15 e Å^−3^

### 

Data collection: *APEX2* (Bruker, 2005 ▶); cell refinement: *SAINT* (Bruker, 2005 ▶); data reduction: *SAINT*; program(s) used to solve structure: *SHELXS97* (Sheldrick, 2008 ▶); program(s) used to refine structure: *SHELXL97* (Sheldrick, 2008 ▶); molecular graphics: *ORTEP-3 for Windows* (Farrugia, 1997 ▶); software used to prepare material for publication: *WinGX* (Farrugia, 1999 ▶).

## Supplementary Material

Crystal structure: contains datablocks I, global. DOI: 10.1107/S1600536811016941/cv5077sup1.cif

Structure factors: contains datablocks I. DOI: 10.1107/S1600536811016941/cv5077Isup2.hkl

Supplementary material file. DOI: 10.1107/S1600536811016941/cv5077Isup3.cml

Additional supplementary materials:  crystallographic information; 3D view; checkCIF report

## Figures and Tables

**Table 1:** Hydrogen-bond geometry (Å, °)

*D*—H⋯*A*	*D*—H	H⋯*A*	*D*⋯*A*	*D*—H⋯*A*
C17—H17⋯O1^i^	0.93	2.51	3.383 (3)	156

Symmetry code: (i) 
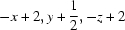
.

**Fig. 1.**
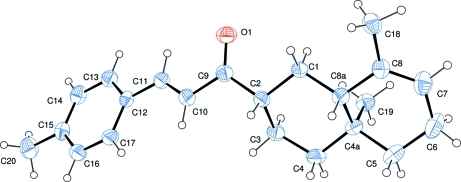
Molecular structure of the title compound with the atom-labelling scheme. Displacement ellipsoids are drawn at the 30% probability level. H atoms are represented as small spheres of arbitrary radii.

**Fig. 2.**
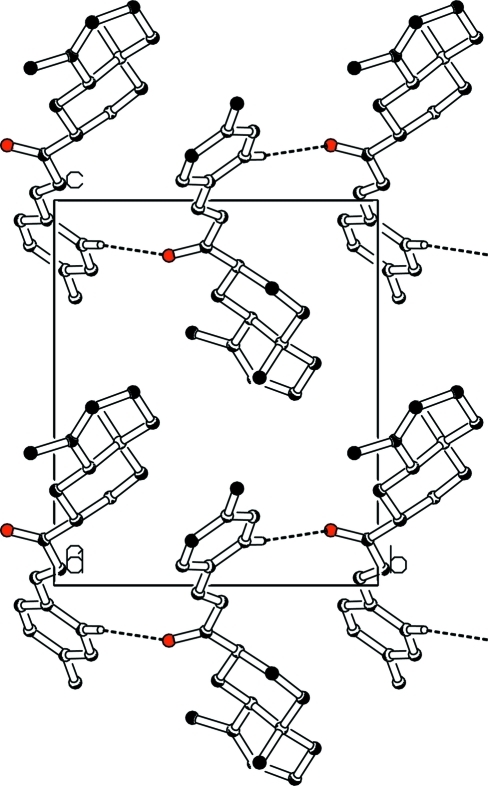
A portion of the crystal packing showing hydrogen-bonded (dashed lines) chains of the molecules. H atoms not involved in hydrogen bonding have been omitted for clarity.

## supplementary crystallographic information

### Comment

Our work lies within the framework of the valorization of medicinal plants and concerns Inula Viscosa (*L*) Aiton or Dittrichia Viscosa (*L*) Greuter. This plant is widespread in Mediterranean area and extends to the Atlantic cost of Morocco. It is a well known medicinal plant (Shtacher & Kasshman, 1970; Bohlmann & Gupta, 1982), which exhibits some pharmacological activities (Azoulay *et al.*, 1986). This plant has been the subject of chemical investigation in terms of isolating sesquiterpene lactones (Bohlmann *et al.*, 1977) and sesquiterpene acids (Ceccherelli *et al.*, 1988). The isocostic acid is a major constituent of the dichloromethane extract of the Inula viscosa (*L*). The literature does not report any results on the transformation of this acid. In order to prepare products with high added value, we studied the reactivity of this acid. Thus, from this acid, we have prepared by reaction of Curtius the 1 - (4a, 8dimethyl-1,2,3,4, 4a,5,6,8a-octahydronaphthalen-2-yl)-ethanone, which was synthesized by Kutney *et al.* (1984). The condensation of this ketone with *para*- methylbenzaldehyde in the presence of sodium hydroxide allowed us to obtain the title compound (I) with a good yield of 80%. The structure of this new derivative of isocostic acid was established by NMR spectral analysis of 1H, 13 C and mass spectroscopy and confirmed by its single-crystal X-ray structure.

The molecule of (I) is built up from two fused six-membered rings, substituted at position 2 by 4-methylphenylpropenoyl group The molecular structure of (I), Fig.1, shows the cyclohexane ring to adopt a chair conformation, as indicated by the total puckering amplitude QT = 0.5617 (17)Å and spherical polar angle θ =7.25 (17)° with φ = 260.6 (14)°. While the cyclohexene ring has a half chair conformation with QT = 0.5071 (18) Å, θ =49.7 (2)°, φ = 12.5 (6)° (Cremer & Pople, 1975). In the crystal structure, weak intermolecular C—H···O hydrogen bonds (Table 1) link molecules into chains in [010] (Fig. 2).

### Experimental

In a flask was introduced a mixture of 500 mg (2.42 mmol), of 1 - (4a, 8-dimethyl-1, 2,3,4,4a,5,6,8a-octahydronaphthalen-2-yl)-ethanone, 257 mg (2.42 mmol.) of *para* chlor-benzaldehyde, 30 ml of anhydrous ethanol and 1 ml of a solution of sodium hydroxide(2 N). The mixture was stirred for three hours at room temperature. After neutralization followed by extraction three time with 20 ml of dichloromethane, the organic phase is dried over sodium sulfate, then evaporated under vacuum. Chromatography on a column of silica gel with hexane-ethyl acetate (97/3) as eluent of the residue allowed us to obtain 3-(4-méthylphenyl)-1-(4a, 8-dimethyl-1,2,3,4,4a, 5,6,8a-octahydronaphthalen-2-yl)prop-2-en-1-one with a yield of 80%. The title compound is recrystallized in hexane-ethyl acetate (70/30).

### Refinement

All H atoms were fixed geometrically and treated as riding with C—H = 0.93 Å(aromatic), 0.96Å (methyl), 0.97 Å (methylene), 0.98Å (methine) with *U*_iso_(H) = 1.2U_eq_ (aromatic, methylene, methine) or *U*_iso_(H) = 1.5*U*_eq_ (methyl). In the absence of significant anomalous scatterers, the absolute configuration could not be reliably determined, so 1566 Friedel pairs were merged before the final refinement.

### Crystal data

**Table:**

C_22_H_28_O	*F*(000) = 336
*M**_r_* = 308.44	*D*_x_ = 1.124 Mg m^−^^3^
Monoclinic, *P*2_1_	Mo *K*α radiation, λ = 0.71073 Å
Hall symbol: P2yb	Cell parameters from 8379 reflections
*a* = 7.1577 (2) Å	θ = 3.2–26.4°
*b* = 10.3456 (2) Å	µ = 0.07 mm^−^^1^
*c* = 12.3663 (3) Å	*T* = 298 K
β = 95.557 (1)°	Box, colourless
*V* = 911.43 (4) Å^3^	0.37 × 0.16 × 0.16 mm
*Z* = 2	

### Data collection

**Table:**

Bruker X8 APEXII CCD area-detector diffractometer	1834 reflections with *I* > 2σ(*I*)
Radiation source: fine-focus sealed tube	*R*_int_ = 0.020
graphite	θ_max_ = 26.4°, θ_min_ = 3.2°
φ and ω scans	*h* = −8→8
8379 measured reflections	*k* = −12→10
1957 independent reflections	*l* = −15→15

### Refinement

**Table:**

Refinement on *F*^2^	Secondary atom site location: difference Fourier map
Least-squares matrix: full	Hydrogen site location: inferred from neighbouring sites
*R*[*F*^2^ > 2σ(*F*^2^)] = 0.039	H-atom parameters constrained
*wR*(*F*^2^) = 0.102	*w* = 1/[σ^2^(*F*_o_^2^) + (0.0586*P*)^2^ + 0.0808*P*] where *P* = (*F*_o_^2^ + 2*F*_c_^2^)/3
*S* = 1.11	(Δ/σ)_max_ < 0.001
1957 reflections	Δρ_max_ = 0.16 e Å^−^^3^
212 parameters	Δρ_min_ = −0.15 e Å^−^^3^
1 restraint	Extinction correction: *SHELXL97* (Sheldrick, 2008), Fc^*^=kFc[1+0.001xFc^2^λ^3^/sin(2θ)]^-1/4^
Primary atom site location: structure-invariant direct methods	Extinction coefficient: 0.114 (14)

### Special details

**Table:**

Geometry. All s.u.'s (except the s.u. in the dihedral angle between two l.s. planes) are estimated using the full covariance matrix. The cell s.u.'s are taken into account individually in the estimation of s.u.'s in distances, angles and torsion angles; correlations between s.u.'s in cell parameters are only used when they are defined by crystal symmetry. An approximate (isotropic) treatment of cell s.u.'s is used for estimating s.u.'s involving l.s. planes.
Refinement. Refinement of *F*^2^ against ALL reflections. The weighted *R*-factor *wR* and goodness of fit *S* are based on *F*^2^, conventional *R*-factors *R* are based on *F*, with *F* set to zero for negative *F*^2^. The threshold expression of *F*^2^ > 2σ(*F*^2^) is used only for calculating *R*-factors(gt) *etc*. and is not relevant to the choice of reflections for refinement. *R*-factors based on *F*^2^ are statistically about twice as large as those based on *F*, and *R*- factors based on ALL data will be even larger.

### Fractional atomic coordinates and isotropic or equivalent isotropic displacement parameters (Å^2^)

**Table:**

	*x*	*y*	*z*	*U*_iso_*/*U*_eq_	
C8A	0.4720 (3)	0.6078 (2)	0.69374 (15)	0.0458 (4)	
H1	0.4204	0.6593	0.7503	0.055*	
C4A	0.5857 (3)	0.70276 (19)	0.62901 (16)	0.0479 (5)	
C9	0.8915 (3)	0.4685 (2)	0.88054 (17)	0.0492 (5)	
C1	0.5998 (3)	0.5063 (2)	0.75326 (15)	0.0452 (4)	
H1B	0.5250	0.4505	0.7951	0.054*	
H1A	0.6555	0.4531	0.7003	0.054*	
C2	0.7557 (3)	0.5682 (2)	0.82916 (16)	0.0471 (4)	
H2	0.6963	0.6105	0.8880	0.057*	
C12	1.3719 (3)	0.4780 (2)	1.04958 (14)	0.0447 (4)	
C8	0.3045 (3)	0.5531 (2)	0.62379 (16)	0.0496 (5)	
C10	1.0489 (3)	0.5164 (2)	0.95720 (16)	0.0518 (5)	
H10	1.0404	0.5971	0.9894	0.062*	
C11	1.2020 (3)	0.4446 (2)	0.98015 (15)	0.0493 (5)	
H11	1.2007	0.3633	0.9480	0.059*	
C4	0.7317 (4)	0.7683 (2)	0.70850 (19)	0.0598 (6)	
H4A	0.6678	0.8192	0.7595	0.072*	
H4B	0.8056	0.8269	0.6687	0.072*	
C15	1.7055 (3)	0.5343 (2)	1.18078 (17)	0.0551 (5)	
C19	0.6825 (3)	0.6347 (2)	0.53984 (17)	0.0555 (5)	
H19C	0.7554	0.6962	0.5038	0.083*	
H19A	0.5894	0.5971	0.4881	0.083*	
H19B	0.7634	0.5679	0.5716	0.083*	
C14	1.6908 (3)	0.4253 (3)	1.11618 (17)	0.0579 (6)	
H14	1.7930	0.3699	1.1158	0.069*	
C13	1.5268 (3)	0.3968 (2)	1.05189 (16)	0.0536 (5)	
H13	1.5202	0.3222	1.0097	0.064*	
C17	1.3856 (3)	0.5877 (2)	1.11495 (17)	0.0523 (5)	
H17	1.2836	0.6433	1.1155	0.063*	
C18	0.2247 (3)	0.4273 (3)	0.65615 (18)	0.0600 (6)	
H18A	0.1128	0.4086	0.6096	0.090*	
H18C	0.1951	0.4323	0.7301	0.090*	
H18B	0.3151	0.3598	0.6496	0.090*	
C16	1.5503 (3)	0.6143 (2)	1.17911 (18)	0.0595 (6)	
H16	1.5569	0.6881	1.2223	0.071*	
C7	0.2281 (3)	0.6174 (3)	0.5375 (2)	0.0688 (7)	
H7	0.1239	0.5806	0.4984	0.083*	
C5	0.4492 (4)	0.8042 (2)	0.5771 (2)	0.0683 (7)	
H5B	0.3912	0.8495	0.6338	0.082*	
H5A	0.5187	0.8670	0.5386	0.082*	
C3	0.8631 (3)	0.6731 (2)	0.77156 (19)	0.0602 (6)	
H3B	0.9445	0.7200	0.8253	0.072*	
H3A	0.9417	0.6319	0.7220	0.072*	
C6	0.2963 (4)	0.7440 (3)	0.4981 (3)	0.0849 (9)	
H6A	0.1913	0.8034	0.4874	0.102*	
H6B	0.3451	0.7313	0.4284	0.102*	
C20	1.8842 (4)	0.5651 (4)	1.2517 (2)	0.0800 (8)	
H20A	1.9096	0.6560	1.2482	0.120*	
H20C	1.9867	0.5176	1.2265	0.120*	
H20B	1.8697	0.5412	1.3254	0.120*	
O1	0.8777 (2)	0.35492 (17)	0.85675 (15)	0.0659 (5)	

### Atomic displacement parameters (Å^2^)

**Table:**

	*U*^11^	*U*^22^	*U*^33^	*U*^12^	*U*^13^	*U*^23^
C8A	0.0479 (10)	0.0461 (10)	0.0443 (9)	0.0035 (8)	0.0090 (7)	−0.0006 (8)
C4A	0.0543 (11)	0.0386 (10)	0.0515 (10)	0.0026 (8)	0.0083 (8)	0.0041 (8)
C9	0.0482 (10)	0.0496 (12)	0.0498 (10)	−0.0066 (9)	0.0054 (8)	0.0017 (9)
C1	0.0476 (10)	0.0413 (10)	0.0461 (9)	−0.0071 (8)	0.0016 (7)	0.0033 (8)
C2	0.0484 (10)	0.0463 (11)	0.0461 (9)	−0.0050 (9)	0.0020 (8)	−0.0024 (8)
C12	0.0469 (9)	0.0465 (10)	0.0408 (8)	−0.0001 (8)	0.0049 (7)	0.0069 (8)
C8	0.0400 (9)	0.0596 (12)	0.0498 (10)	0.0045 (9)	0.0070 (8)	0.0004 (9)
C10	0.0505 (10)	0.0529 (12)	0.0511 (10)	−0.0025 (9)	0.0006 (8)	−0.0003 (9)
C11	0.0531 (11)	0.0491 (12)	0.0459 (9)	−0.0033 (9)	0.0053 (8)	0.0019 (9)
C4	0.0737 (14)	0.0397 (11)	0.0655 (12)	−0.0098 (10)	0.0052 (11)	−0.0006 (10)
C15	0.0493 (11)	0.0641 (14)	0.0510 (10)	0.0016 (10)	0.0004 (8)	0.0092 (10)
C19	0.0603 (12)	0.0529 (13)	0.0552 (11)	−0.0008 (10)	0.0150 (9)	0.0040 (10)
C14	0.0507 (11)	0.0678 (15)	0.0560 (11)	0.0154 (11)	0.0089 (9)	0.0068 (11)
C13	0.0592 (12)	0.0517 (12)	0.0509 (10)	0.0087 (10)	0.0102 (8)	−0.0020 (9)
C17	0.0513 (11)	0.0448 (11)	0.0595 (11)	0.0079 (9)	−0.0010 (8)	0.0000 (9)
C18	0.0480 (11)	0.0705 (15)	0.0607 (12)	−0.0073 (10)	0.0009 (9)	−0.0006 (11)
C16	0.0622 (13)	0.0526 (12)	0.0614 (12)	0.0019 (11)	−0.0054 (10)	−0.0058 (10)
C7	0.0499 (12)	0.0862 (18)	0.0685 (14)	0.0054 (12)	−0.0034 (10)	0.0105 (13)
C5	0.0746 (15)	0.0512 (13)	0.0798 (15)	0.0151 (12)	0.0116 (12)	0.0171 (12)
C3	0.0608 (12)	0.0517 (12)	0.0655 (12)	−0.0199 (10)	−0.0073 (10)	0.0052 (11)
C6	0.0705 (16)	0.090 (2)	0.0911 (19)	0.0136 (16)	−0.0063 (14)	0.0377 (17)
C20	0.0566 (13)	0.098 (2)	0.0809 (16)	0.0032 (14)	−0.0141 (12)	0.0002 (16)
O1	0.0613 (9)	0.0470 (9)	0.0866 (11)	−0.0043 (7)	−0.0080 (8)	0.0008 (8)

### Geometric parameters (Å, °)

**Table:**

C8A—C8	1.518 (3)	C15—C16	1.385 (3)
C8A—C1	1.534 (3)	C15—C20	1.513 (3)
C8A—C4A	1.547 (3)	C19—H19C	0.9600
C8A—H1	0.9800	C19—H19A	0.9600
C4A—C4	1.523 (3)	C19—H19B	0.9600
C4A—C19	1.530 (3)	C14—C13	1.384 (3)
C4A—C5	1.532 (3)	C14—H14	0.9300
C9—O1	1.213 (3)	C13—H13	0.9300
C9—C10	1.485 (3)	C17—C16	1.383 (3)
C9—C2	1.514 (3)	C17—H17	0.9300
C1—C2	1.528 (3)	C18—H18A	0.9600
C1—H1B	0.9700	C18—H18C	0.9600
C1—H1A	0.9700	C18—H18B	0.9600
C2—C3	1.543 (3)	C16—H16	0.9300
C2—H2	0.9800	C7—C6	1.496 (5)
C12—C13	1.389 (3)	C7—H7	0.9300
C12—C17	1.391 (3)	C5—C6	1.528 (4)
C12—C11	1.460 (3)	C5—H5B	0.9700
C8—C7	1.329 (3)	C5—H5A	0.9700
C8—C18	1.492 (3)	C3—H3B	0.9700
C10—C11	1.332 (3)	C3—H3A	0.9700
C10—H10	0.9300	C6—H6A	0.9700
C11—H11	0.9300	C6—H6B	0.9700
C4—C3	1.523 (3)	C20—H20A	0.9600
C4—H4A	0.9700	C20—H20C	0.9600
C4—H4B	0.9700	C20—H20B	0.9600
C15—C14	1.380 (4)		
			
C8—C8A—C1	114.82 (17)	H19C—C19—H19A	109.5
C8—C8A—C4A	111.57 (16)	C4A—C19—H19B	109.5
C1—C8A—C4A	111.33 (16)	H19C—C19—H19B	109.5
C8—C8A—H1	106.1	H19A—C19—H19B	109.5
C1—C8A—H1	106.1	C15—C14—C13	121.3 (2)
C4A—C8A—H1	106.1	C15—C14—H14	119.3
C4—C4A—C19	109.86 (18)	C13—C14—H14	119.3
C4—C4A—C5	109.59 (19)	C14—C13—C12	120.9 (2)
C19—C4A—C5	109.17 (17)	C14—C13—H13	119.6
C4—C4A—C8A	108.12 (16)	C12—C13—H13	119.6
C19—C4A—C8A	112.23 (17)	C16—C17—C12	120.3 (2)
C5—C4A—C8A	107.82 (17)	C16—C17—H17	119.9
O1—C9—C10	121.1 (2)	C12—C17—H17	119.9
O1—C9—C2	121.63 (19)	C8—C18—H18A	109.5
C10—C9—C2	117.21 (19)	C8—C18—H18C	109.5
C2—C1—C8A	111.96 (16)	H18A—C18—H18C	109.5
C2—C1—H1B	109.2	C8—C18—H18B	109.5
C8A—C1—H1B	109.2	H18A—C18—H18B	109.5
C2—C1—H1A	109.2	H18C—C18—H18B	109.5
C8A—C1—H1A	109.2	C17—C16—C15	121.8 (2)
H1B—C1—H1A	107.9	C17—C16—H16	119.1
C9—C2—C1	111.90 (17)	C15—C16—H16	119.1
C9—C2—C3	110.14 (17)	C8—C7—C6	125.3 (3)
C1—C2—C3	112.23 (15)	C8—C7—H7	117.4
C9—C2—H2	107.4	C6—C7—H7	117.4
C1—C2—H2	107.4	C6—C5—C4A	112.1 (2)
C3—C2—H2	107.4	C6—C5—H5B	109.2
C13—C12—C17	118.05 (19)	C4A—C5—H5B	109.2
C13—C12—C11	119.1 (2)	C6—C5—H5A	109.2
C17—C12—C11	122.84 (19)	C4A—C5—H5A	109.2
C7—C8—C18	120.9 (2)	H5B—C5—H5A	107.9
C7—C8—C8A	120.8 (2)	C4—C3—C2	112.33 (19)
C18—C8—C8A	118.30 (18)	C4—C3—H3B	109.1
C11—C10—C9	120.5 (2)	C2—C3—H3B	109.1
C11—C10—H10	119.7	C4—C3—H3A	109.1
C9—C10—H10	119.7	C2—C3—H3A	109.1
C10—C11—C12	127.9 (2)	H3B—C3—H3A	107.9
C10—C11—H11	116.0	C7—C6—C5	112.6 (2)
C12—C11—H11	116.0	C7—C6—H6A	109.1
C4A—C4—C3	113.21 (18)	C5—C6—H6A	109.1
C4A—C4—H4A	108.9	C7—C6—H6B	109.1
C3—C4—H4A	108.9	C5—C6—H6B	109.1
C4A—C4—H4B	108.9	H6A—C6—H6B	107.8
C3—C4—H4B	108.9	C15—C20—H20A	109.5
H4A—C4—H4B	107.7	C15—C20—H20C	109.5
C14—C15—C16	117.64 (19)	H20A—C20—H20C	109.5
C14—C15—C20	121.4 (2)	C15—C20—H20B	109.5
C16—C15—C20	121.0 (2)	H20A—C20—H20B	109.5
C4A—C19—H19C	109.5	H20C—C20—H20B	109.5
C4A—C19—H19A	109.5		

### Hydrogen-bond geometry (Å, °)

**Table:**

*D*—H···*A*	*D*—H	H···*A*	*D*···*A*	*D*—H···*A*
C17—H17···O1^i^	0.93	2.51	3.383 (3)	156

Symmetry codes: (i) −*x*+2, *y*+1/2, −*z*+2.
